# Contrasting immune responses in COVID-19: insights from healthcare workers and infected patients on plasmablast, pDC, and NK cell dynamics

**DOI:** 10.3389/fimmu.2025.1693903

**Published:** 2026-01-07

**Authors:** Laura Martín-Pedraza, Eulalia Rodríguez-Martín, Erick De La Torre-Tarazona, Elena Moreno, Roberto Pariente-Rodríguez, Paulette Esperanza Walo-Delgado, Javier García-Pérez, Jorge Díaz, Laura Luna, Mario Rodríguez-Dominguez, Juan Carlos Galán, Claudia Geraldine Rita, Ana del Amo-de Palacios, Roser Navarro-Soler, María Fons, José Alcamí, Santiago Moreno, Luisa María Villar, Sergio Serrano-Villar

**Affiliations:** 1 Servicio de Enfermedades Infecciosas, Hospital Ramón y Cajal, IRYCIS, Universidad de Alcalá y CIBER de Enfermedades Infecciosas (CIBERINFEC), Madrid, Spain; 2 Servicio de Inmunología, Hospital Ramón y Cajal, IRYCIS, Universidad de Alcalá, Madrid, Spain; 3 Laboratorio de Referencia e Investigación en Retrovirus, Instituto de Salud Carlos III, CIBER de Enfermedades Infecciosas (CIBERINFEC), Madrid, Spain; 4 Servicio de Microbiología, Hospital Ramón y Cajal, IRYCIS, y CIBER en Epidemiología y Salud Pública (CIBERESP), Madrid, Spain; 5 Facultad de Medicina, Campus de Ciencias de la Vida, Universidad Antonio de Nebrija, Madrid, Spain

**Keywords:** SARS-CoV-2, COVID-19, immune response, plasmablasts, dendritic cells, healthcare workers, NK cells

## Abstract

**Background:**

The COVID-19 pandemic, caused by SARS-CoV-2, has highlighted significant variability in disease severity, ranging from asymptomatic cases to severe acute respiratory distress syndrome.

**Objective:**

Understanding the immune responses that contribute to this variability, particularly among healthcare workers (HCWs) frequently exposed to the virus, is essential.

**Materials and methods:**

This was a prospective single-center longitudinal cohort study. We included hospitalized COVID-19+ patients, classified as having mild or moderate-to-severe symptoms, and unvaccinated HCWs with low susceptibility. Peripheral blood mononuclear cells (PBMCs) were collected and analyzed using flow cytometry. We measured the frequencies of key immune subsets, including plasmablasts, plasmacytoid dendritic cells (pDCs), and Natural Killer (NK) cells. SARS-CoV-2 neutralizing antibodies (NAbs) were quantified using pseudotyped HIV particles.

**Results:**

COVID-19+ patients exhibited a significant increase in plasmablasts, a B-cell subset responsible for producing neutralizing antibodies, which correlated with disease severity (p=0.0082). Conversely, uninfected HCWs had low levels of plasmablasts but significantly higher levels of plasmacytoid dendritic cells (pDCs) (p<0.0001), which produce interferons upon activation by viral antigens. Additionally, HCWs had a higher percentage of CD56bright NK cells than the susceptible patients (p=0.02).

**Conclusion:**

Our findings suggest that immune dysregulation, characterized by increased plasmablasts and reduced pDC and NK cell responses, contributes to COVID-19 severity. Strong pDC and NK cell responses may confer protection against SARS-CoV-2. These insights into immune responses may inform strategies for therapeutic interventions and vaccine development.

## Introduction

The COVID-19 pandemic, caused by SARS-CoV-2, has resulted in widespread morbidity and mortality globally (1–3). Although significant research has been conducted, the mechanisms underlying the variability in disease severity—including why some healthcare workers (HCWs) remained uninfected despite repeated exposure—are not fully understood, partly due to the complex interplay between viral factors and individual immune responses, which remain inadequately characterized. COVID-19 severity ranges from asymptomatic or mild presentations to acute respiratory distress syndrome (ARDS) (4), highlighting the need to clarify host factors, such as genetic predispositions, immune response variability, or environmental exposures, that influence these outcomes. The immune system is central to this variability, acting as either a protective barrier or contributor to severe disease pathology (5). HCWs, frequently exposed to SARS-CoV-2 yet not always infected or severely affected, represent a valuable population for studying immunity to the virus (6–8).

The immune response to SARS-CoV-2 involves multiple components of the innate and adaptive immune systems. A well-coordinated immune response can limit viral spread and promote recovery. In contrast, dysregulated responses can lead to excessive inflammation, often described as a “cytokine storm,” resulting in poor clinical outcomes (9). Previous studies have highlighted several key immune players, including plasmablasts, which are responsible for producing antibodies; plasmacytoid dendritic cells (pDCs), which are crucial for producing type I interferons and initiating antiviral responses; and Natural Killer (NK) cells, which play a role in directly eliminating infected cells, as central to shaping the course of SARS-CoV-2 infection (10, 11). However, the exact contribution of these immune subsets, particularly in determining susceptibility and severity, remains an area of active investigation.

Understanding the immune profiles of unvaccinated HCWs, compared to patients with varying COVID-19 severity, could provide insights into protective versus pathogenic immune mechanisms. Here, we aimed to investigate the immunological features that differentiate individuals who are repeatedly exposed to but remain uninfected from those who develop mild or severe COVID-19. We specifically focused on key immune cell populations such as plasmablasts, pDCs, and NK cells to elucidate their roles in SARS-CoV-2 susceptibility and disease progression.

## Materials and methods

### Participants

We conducted a prospective cohort study at Ramón y Cajal University Hospital, Madrid, Spain. All study visits and blood draws were performed during 2020, in the pre-vaccination period of the pandemic in Spain, Participants were categorized based on their SARS-CoV-2 infection status at the time of sampling into COVID-19+ (further divided into mild (M) or moderate-severe (S) symptoms) and “Controls-LS” (healthcare workers with no infection and low susceptibility (LS) to SARS-CoV-2 infection).

COVID-19+ participants were hospitalized patients with confirmed SARS-CoV-2 infection detected via PCR testing of nasopharyngeal swabs, sputum, or lower respiratory tract secretions within the first 7 days of symptom onset. These patients were then classified according to the severity of their clinical presentation as mild (asymptomatic for a week after diagnosis and without the need for supplemental oxygen), moderate (bilateral radiologic infiltrates or opacities requiring supplemental oxygen), or severe (acute respiratory distress syndrome).

The “Controls-LS” group included unvaccinated, asymptomatic healthcare workers who had negative PCR tests for SARS-CoV-2 in their nasopharyngeal exudate and reported at least three high-risk exposures to SARS-CoV-2 without experiencing symptoms suggestive of infection (12). They were persistently negative for SARS-CoV-2 by PCR testing and did not have detectable SARS-CoV-2 IgM or IgG in plasma. Most exposures involved a lack of protection during aerosol-generating activities, close contact with patients without wearing facemasks, and close contact with confirmed COVID-19 cases. All healthcare workers remained PCR- and seronegative for SARS-CoV-2 during the recruitment period and the subsequent months. Long-term follow-up was not feasible, as several participants changed workplace and vaccination campaigns were initiated soon after the study period.

This study was approved by the Ethics Committee of Ramón y Cajal University Hospital (095/20). All participants provided written informed consent before participation.

### Sample collection

Study samples were obtained at hospital admission, before initiation of specific COVID-19 therapy, including corticosteroids, tocilizumab, or other immunomodulatory drugs. Heparinized whole blood samples were collected from each patient immediately upon hospital admission, during 2020. Peripheral blood mononuclear cells (PBMCs) were isolated within 2 hours by Ficoll-Histopaque density gradient centrifugation (Life Technologies Ltd, UK) and stored in liquid nitrogen in aliquots of 5–6 × 10^6 cells until further analysis.

### Monoclonal antibodies

The following monoclonal antibodies were used: CD8-FITC, CD24-FITC, CD57-FITC, CD16-FITC, Perforin-FITC, IL-1beta-FITC, IFN-gamma-FITC, CD197-PE (CCR7-PE), CD27-PE, CD366-PE (TIM-3-PE), CD19-PE, CD3-PE, CD56-PE, Granzyme-B-PE, IL-10-PE, GMCSF-PE, CD3-PerCP, CD38-PE-Cy5, CD28-PE-Cy5, CD11c-PE-Cy5, TNFalpha-PerCP-Cy5.5, CD25-PE-Cy7, CD19-PE-Cy7, CD274-PE-Cy7 (PD-L1)-PE-Cy7, CD56-PE-Cy7, CD45RO-APC, CD86-APC, CD3-APC, CD123-APC, CD158a-APC, CD4-APC-H7, CD8-APC-H7, CD14-APC-H7, CD127-BV421, CD80-BV421, CD152-BV421, DR-v450, CD158b-BV421, IL-6-BV421, CD3-BV421, CD45-V500-C (all from BD Biosciences, San Diego, CA), and IL17-APC (R&D Systems).

### Labeling of surface antigens

Blood leukocyte subsets were studied using flow cytometry as previously described (13). Briefly, aliquots of 10^6 PBMCs were resuspended in RPMI 1640 medium (Thermofisher Scientific) plus 2 mM L-glutamine and 10% fetal bovine serum (complete medium), stained with monoclonal antibodies for 30 minutes at 4°C in the dark, washed twice with PBS, and analyzed using a FACSCanto II flow cytometer (BD Biosciences) within 1 hour of staining.

### Flow cytometry

**Figure 1:**
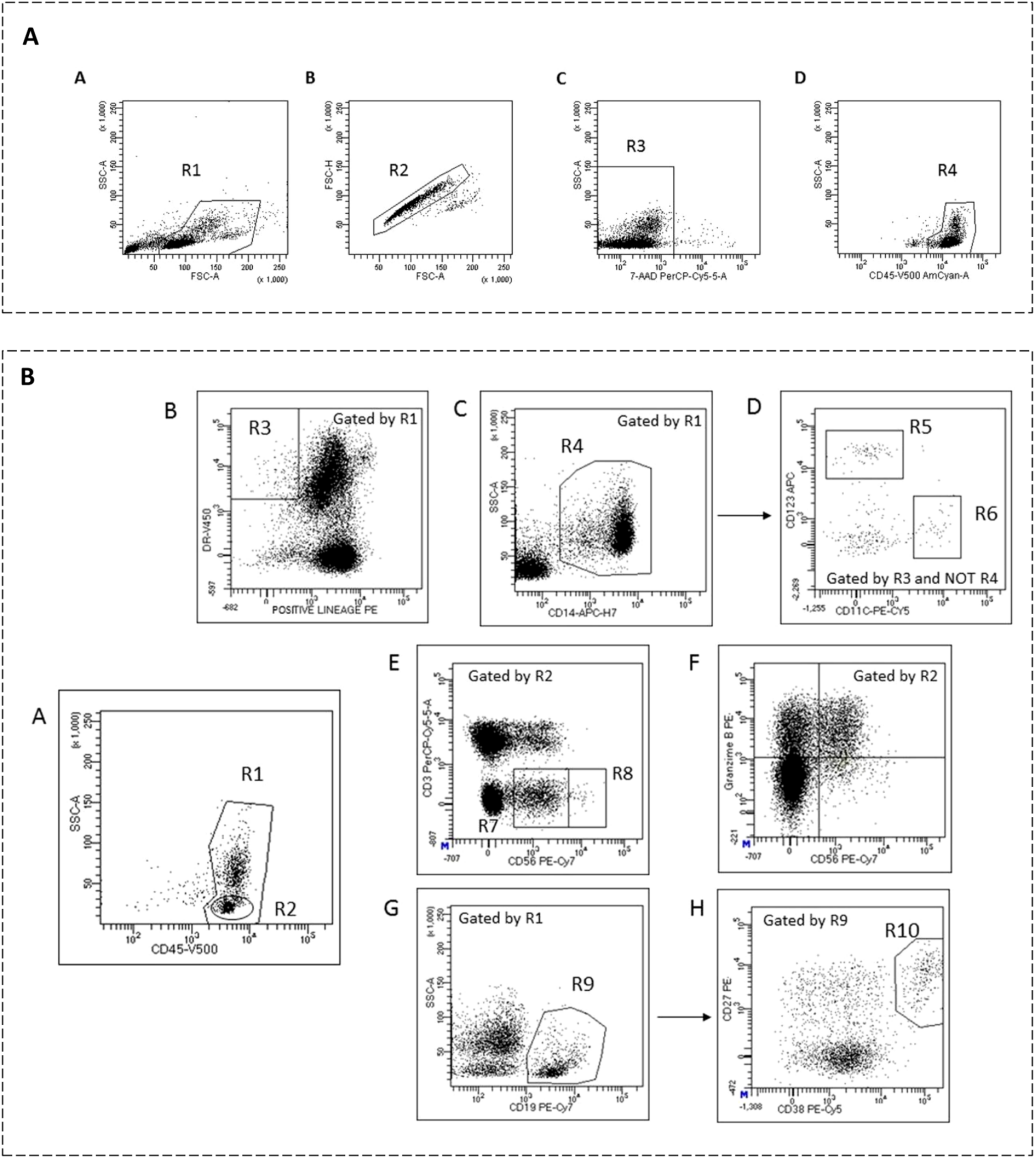
**(A)** Representative images of gating strategy for flow cytometry analysis. Cells were gated to exclude debris (A, gate R1). Single cells (B, gate R2) were further analyzed to establish viable cells by non-staining with 7-AAD (C, gate R3). CD45 staining was used to identify peripheral blood mononuclear cells (PBMCs) (D, gate R4) including lymphocytes and monocytes. **(B)** Gating strategy employed for the identification of PBMC subsets by flow cytometry. Total events were first gated to exclude debris, duplets, and nonviable cells using 7-AAD, main region was set around viable cells expressing intermediate to high CD45 with low to intermediate side scatter to select total PBMC (A, gate R1) and total lymphocytes (A, gate R2). Expression of HLA-DR (B, gate R3) and exclusion of lineage-positive (CD19+, CD3+, and CD56+) cells and monocytes (CD14high expressing cells, C, gate R4) identified plasmacytoid (CD123+ expressing cells, D, gate R5) and myeloid (CD11c+ expressing cells, D, gate R6) dendritic cells. Lymphocytes expressing CD56+ (NK cells, E) were subdivided into CD3- CD56dim cells (gate R7) and CD3- CD56bright cells (gate R8). NK cells expressing granzyme B are shown in plot (F) B cells were identified by their CD19 expression (G, gate R9) and gated for CD27++ and CD38++ expressing cells that identified plasmablast subpopulation (H, gate R10).

Data analysis was performed using FACSDIVA software V.9.0 (BD Biosciences). Gating was performed to include lymphocytes and monocytes, while excluding debris and apoptotic cells; a minimum of 5 × 10^4 events were analyzed. Representative images of the gating strategy used for flow cytometry analysis are shown in **Figure 1**. Mean autofluorescence values were set using appropriate negative isotype controls. CD4+ and CD8+ T cells were classified as: naive (CCR7+ CD45RO–), central memory (CM) (CCR7+ CD45RO+), effector memory (EM) (CCR7–CD45RO+), or terminally differentiated (TD) (CCR7– CD45RO–). Regulatory CD4 T cells (Treg) were defined as CD3+ CD4+ CD25hi CD127low. Senescent CD4+ and CD8+ T cells were defined as CD57+CD28–. B cells were classified as: naive (CD19+ CD38dim CD27–), memory (CD19+ CD27dim CD38dim), plasmablasts (CD19+ CD27hi CD38hi), transitional B cells (CD19+ CD27– CD24hi CD38hi), or regulatory B cells (Breg) (CD19+ IL10+). PD-L1 expression was explored in monocytes by studying its co-expression with CD14 in PBMCs. CD56+ cells were subdivided into natural killer subsets. For intracellular cytokine staining, non-stimulated PBMCs and appropriate isotype controls were used (**Supplementary Figures S1**, **2**).

We explored the intracellular production of IL1β, IL6, IL10, and TNFα by monocytes; IFNγ, GMCSF, TNFα, and IL17 by CD4 and CD8 T cells; and perforin and granzyme-B by NK cells. For intracellular cytokine production by T cells, aliquots containing 1 x 106 PBMCs were suspended in 1 ml of complete medium supplemented with 50 ng/ml phorbol 12- myristate 13-acetate (PMA) (Sigma–Aldrich) and 750 ng/ml ionomycin (Sigma–Aldrich). Additionally, 2 μg/ml brefeldin A (GolgiPlug, BD Biosciences) and 2.1 μM Monesin (GolgiStop, BD Biosciences) were added. The suspension was incubated in polypropylene tubes at 37°C in a 5% CO2 atmosphere for 4 h. Following the incubation period, the PBMCs were washed with phosphate-buffered saline (PBS), resuspended and stained in the dark for 30 min at 4°C with appropriate amounts of monoclonal antibodies targeting surface antigens. The cells were subsequently washed with PBS, fixed, and permeabilized for 20 min at 4°C in the dark using a Cytofix/Cytoperm Kit (BD Biosciences). After two washes with Perm/Wash solution (BD Biosciences), the cells were stained intracellularly for 30 min at 4°C in the dark with monoclonal antibodies specific to the cytokines mentioned above. Two additional washes were subsequently performed, and the PBMCs were analyzed by flow cytometry. Nonstimulated PBMCs were used as controls for basal production. For perforin and granzyme -N production by NK cells, no stimulation was performed. We also explored intracellular cytokine production by monocytes by stimulating aliquots of 106 PBMCs with 1 mg/mL lipopolysaccharide (from Escherichia coli O111: B4; Merck, Kenilworth, NJ) during 4 hours at 37°C in 5% CO2.We recorded the percentages of each leukocyte subset over total mononuclear cells (PBMCs), also the percentages of each T, B, NK subpopulation, and monocyte population relative to their respective total cell types.

### Neutralizing antibodies

SARS-CoV-2 neutralizing antibodies (NAbs) were determined using HIV particles pseudotyped with the SARS-CoV-2 Spike protein, as previously described (14). Briefly, HIV pseudoviruses carrying the renilla-luciferase reporter were prepared by co-transfecting HEK-293T cells with the pNL4-3ΔenvRen backbone (15) and pcDNA3.1-S-COV-2Δ19-G614 (HIV_SARS-CoV-2) using the calcium phosphate method. Forty-eight hours post-transfection, cell culture supernatants were collected, and the p24 antigen was titrated using electrochemiluminescence immunoassay (Roche Diagnostics).

Neutralization assays were performed in fourfold serial dilutions of heat-inactivated plasma (1:32-1:131072), which were preincubated with pseudoviruses (~10 ng p24 Gag/well of HIV_SARS-CoV-2) for 1 hour at 37°C. Subsequently, VeroE6 cells were added. At 48 hours post-infection, cells were lysed and viral infectivity was assessed by measuring luciferase activity (Renilla Luciferase Assay; Promega) using a 96-well plate luminometer. NAbs titers were calculated as the neutralizing titer 50 (NT50), defined as the highest dilution of serum resulting in a 50% reduction of luminescence compared to the control without serum. Pseudoviruses expressing VSV-G protein instead of the SARS-CoV-2 spike were used as a specificity control. NT50 < 32 was considered absence of neutralization, indicated with a value of 16. NT50 values were summarized as median and interquartile range (IQR).

### Bioinformatic statistical analysis

Non-parametric tests, including the Mann-Whitney U test and Spearman correlation, were selected for analyzing immunological populations and neutralizing antibodies due to the non-normal distribution of the data. We fitted adjusted linear regression models to account for potential confounders, including sex, age, diabetes mellitus, and hypertension.

We performed UMAP analyses using the umap package to reduce dimensionality and visualize patterns in the dataset. Distance matrices were computed using Spearman correlation (vegan package), and clustering was performed with Gaussian mixture models implemented in the mclust package. Data visualization was achieved with ggplot2, enhanced with ggforce for cluster delineation and ggsci for applying D3-inspired color scales. We generated heatmaps using the pheatmap package to visualize the scaled data (scaled by columns), incorporating row annotations derived from metadata.

Statistical and visualization analyses were performed using GraphPad Prism 9.0 (GraphPad Prism Inc.) and R software (Versión 2024.09.0 + 375, R Core Team, 2024).

## Results

### Study population

**Table 1** presents the general characteristics of study participants, including 28 low-susceptible controls (Control-LS) and 37 patients with early-onset COVID-19, stratified into mild and moderate-severe cases. As anticipated, older age, male sex, diabetes, and hypertension were associated with increased disease severity.

**Table 1:** General characteristics of the study population.

Factor		Controls-LS	Mild	Moderate-severe	p-value*
N		28	10	27	
Age, median (IQR)		43 (36, 51)	56 (50, 75)	74 (8, 880)	<0.001
Sex	Woman	22 (79%)	5 (50%)	10 (37%)	0.007
	Man	6 (21%)	5 (50%)	17 (63%)	
Symp. initition, median (IQR)		NA	6.5 (2.5, 9.5)	4 (2, 7)	0.29
HTA	No	27 (96%)	4 (40%)	13 (48%)	<0.001
	Yes	1 (4%)	6 (60%)	14 (52%)	
DM	No	28 (100%)	10 (100%)	23 (88%)	0.010
	Yes	0 (0%)	0 (0%)	3 (12%)	
Obesity	No	26 (93%)	7 (70%)	22 (81%)	0.19
	Yes	2 (7%)	3 (30%)	5 (19%)	
Lung Disease	No	26 (93%)	9 (90%)	23 (85%)	0.65
	Yes	2 (7%)	1 (10%)	4 (15%)	
Immunosupresors	No	NA	9 (90%)	23 (92%)	0.85
	Yes	NA	1 (10%)	2 (8%)	
D-Dimer (ng/ml), median (IQR)		NA	1267 (872, 3236)	843 (551, 1745)	0.27
C reactive protein (mg/L), median (IQR)		NA	15.5 (5.4, 54.2)	64.3 (23.1, 206.1)	0.053
WBC (abs. number) 10^3/µL, median (IQR)		NA	7.1 (6.1, 8.9)	7.0 (5.5, 11)	0.76
Lymphocyte (abs. number) 10^3/µL, median (IQR)		NA	2.3 (1.6, 2.6)	1.0 (0.7, 1.3)	<0.001

*Comparison between Moderate-Severe COVID-19 patients and Controls-LS adults.

### Phenotypic characterization of subpopulations and functional studies

**Figure 2:**
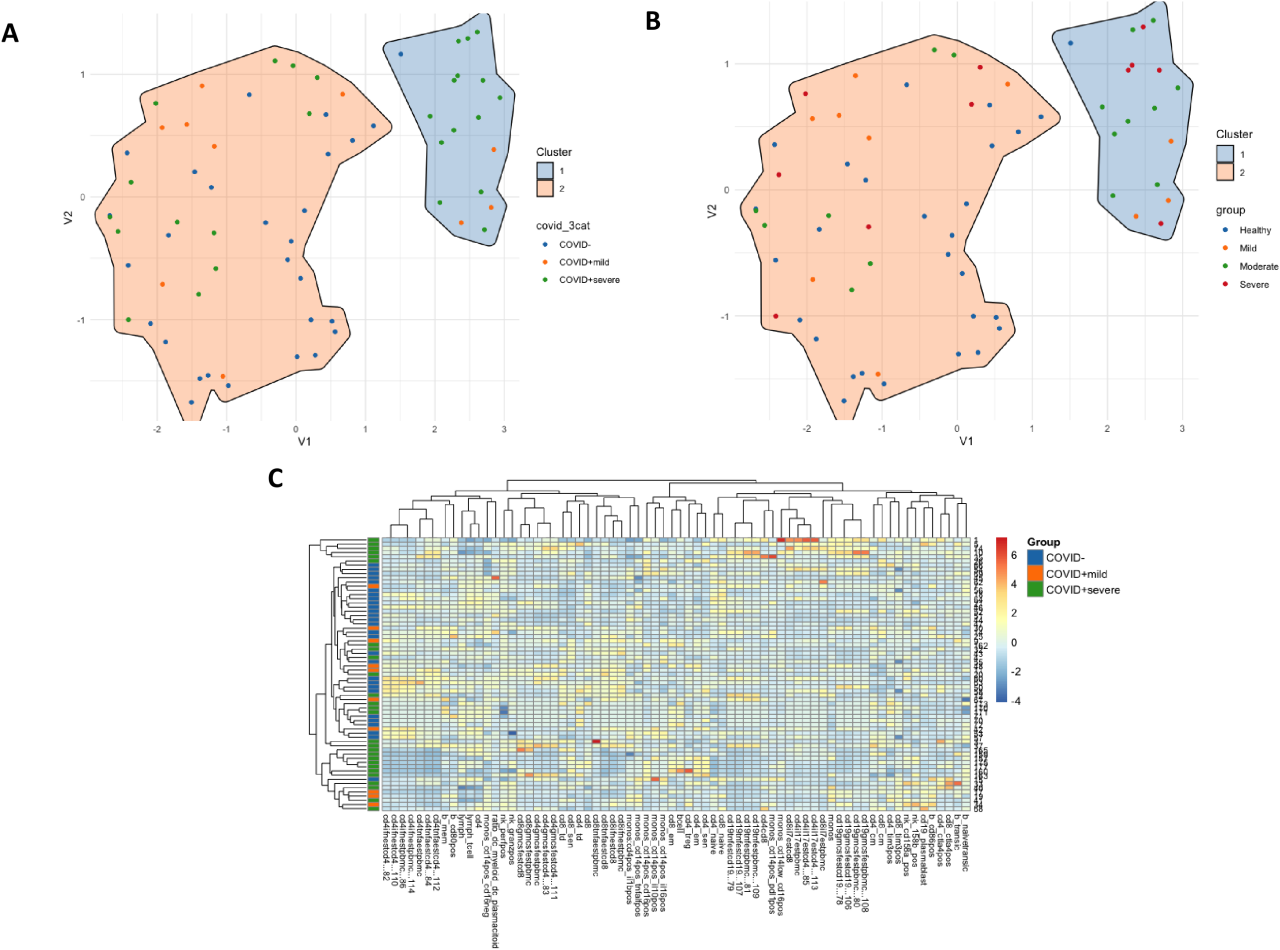
**(A)** A two-dimensional UMAP projection illustrates the immunological landscape of individuals with varying severities of COVID-19 infection. Two-dimensional UMAP projection illustrating the global immunological landscape of study participants based on integrated phenotypic and functional flow-cytometry parameters. Each point represents an individual participant; axes (V1 and V2) summarize multidimensional immunological features reduced to two dimensions for visualization. Participants are color-coded according to group: healthcare workers with low susceptibility (Controls-LS, blue), mild COVID-19 (orange), moderate and sever COVID-19 (green). **(B)** Similar representation of UMAP analysis where participants are grouped as to group: Controls-LS, blue, mild COVID-19 (orange), moderate COVID-19 (green), and severe COVID-19 (red). Each point represents an individual patient, with the axes (V1 and V2) summarizing complex immunological parameters reduced to two dimensions for visualization purposes. Patients are color-coded according to the severity of their infection: healthcare workers (Controls_LS) are in blue, mild cases (COVID19+_mild) are in orange, and a third category (COVID19+_severe) is in green. Two main clusters are highlighted by convex hulls, demonstrating distinct immunological profiles. The smaller cluster predominantly consists of severe cases, while the larger cluster includes mild cases and the controls_LS indicating potential differences in immunological response related to the disease severity. The separation between clusters suggests a significant divergence in the immunological parameters measured, which may correlate with the clinical manifestations of the disease. **(C)** Unsupervised hierarchical clustering heatmap showing the normalized (z-score) frequency of phenotypic and functional immune markers across all participants. Each column represents a participant, and each row represents a specific immunological parameter. The color gradient reflects relative abundance (red = high, blue = low). Dendrograms represent clustering of participants (top) and immune markers (left). Moderate and severe patients are interspersed, confirming overlapping immune profiles, whereas mild cases and Controls-LS cluster more closely together.

We performed UMAP analysis based on the phenotypic and functional flow cytometry analysis to discern whether the overall immunologic features could differentiate the study groups. The analysis suggested that Controls-LS and mild cases are immunologically more similar than the more severe cases. The analysis suggested that Controls-LS and mild cases are immunologically more similar than the more severe cases (**Figure 2A**). In a revised analysis, patients were stratified into mild, moderate, and severe categories. Two main clusters were still observed, but these were not explained by disease severity or baseline biochemical parameters, suggesting inter-individual immune heterogeneity (**Figure 2B**). Consistent with the UMAP projection, the hierarchical clustering heatmap revealed that moderate and severe patients did not segregate into distinct groups. Instead, these patients were interspersed across clusters, indicating substantial overlap in immunophenotypic and functional profiles. In contrast, low-susceptible controls and mild cases clustered more closely together, suggesting a shared immune landscape among less severe or uninfected individuals (**Figure 2C**).

### Immunological characterization and SARS-CoV-2 susceptibility

We analyzed phenotypic and functional flow cytometry profiles to determine differences across groups. As mentioned in Material and Methods section, all patient’ samples analyses reflect the baseline immune profile before treatment or clinical progression.

Controls-LS exhibited significant differences compared to COVID-19+ cases in various immune cell subsets, including T and B lymphocytes, CD4 and CD8 maturation markers, and markers of cellular senescence and inflammation. Detailed distributions for each immune subset across study groups are provided in **Supplementary Figures S3–S10**.

**Figure 3:**
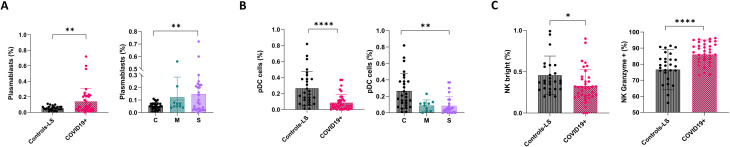
Principle immunological populations: Plasmablast **(A)**, Plasmacytoid Dendritic cells **(B)** and Natural Killer cells **(C)**. Patients are divided according to the severity of their infection: Controls_LS (C) are in gray with vertical line pattern, COVID19+ patients in pink with obliquus line pattern, divided into mild COVID19+ cases (M) in green with dots pattern, and moderate-severe COVID19+ cases (S) in purple with no fill pattern. Data were analyzed by T-Test and one-way ANOVA, mean ± SEM (*p < 0.05, **p < 0.01, ****p< 0.0001).

Among immune subsets, plasmablasts exhibited the clearest differences across groups. COVID-19 patients demonstrated a marked expansion of plasmablasts, showing a threefold increase compared to the Control-LS group (15%; median of 0.070, IQR: 0.03-0.21 vs. 5%; median of 0.05, IQR: 0.032-0.07, p=0.0082) (**Figure 3A**). The plasmablast frequency correlated directly with disease severity, with higher values in severe cases than mild ones (0.15% vs. 0.12%, respectively), however, no differences in % plasmablast among COVID-19-positive patients compared to Control-LS individual divided by sex (p=0.0843) (**Supplementary Figure S11A**). Adjusted linear regression analyses for sex, age, diabetes mellitus, and hypertension showed no significant confounding effects (P-values > 0.45).

Plasmablasts are transient, antibody-secreting cells that emerge from B cells upon antigen exposure, such as to the SARS-CoV-2 spike protein. These cells represent an intermediate phase between activated B cells and mature plasma cells, and they play a crucial role in antibody production during the early adaptive immune response. Correspondingly, the presence of neutralizing antibodies was elevated in COVID-19 patients (median NT50: 130.5, IQR: 16.0-518.8) compared to Control-LS participants, in whom neutralization was largely absent (NT50 < 32; p<0.0001). However, no differences were observed according to the disease severity (**Supplementary Figure S12**). We next assessed whether plasmablast expansion was associated with neutralizing antibody production. A modest but significant positive correlation was observed between plasmablast frequency and SARS-CoV-2 neutralizing antibody titers (Spearman r = 0.34, p = 0.045), suggesting that plasmablast activation reflects the intensity of the humoral response (**Supplementary Figure S13**).

Plasmacytoid dendritic cells (pDCs) also exhibited significant differences between groups. pDCs are pivotal during viral infections, given their capacity to produce type I interferons, and their dysfunction may contribute to severe COVID-19 outcomes (11, 16). In our cohort, Control-LS individuals had significantly higher median pDC frequencies (75% higher; median of 0.20, IQR: 0.097-0.38) compared to COVID-19 patients (median 0.05, IQR: 0.015-0.11, P<0.0001) (**Figure 3B**). No significant sex differences in pDCs frequency were observed within infected or control groups (P-values > 0.40). However, there was a pronounced reduction in pDC frequency among COVID-19-positive women compared to Control-LS women (P<0.0001) (**Supplementary Figure S11B**). Adjusted regression analyses showed hypertension significantly influenced pDC frequency in patients (P = 0.018), whereas sex, age, and diabetes mellitus did not (P-values > 0.25).

### Functional immune profiling

Natural killer (NK) cells, critical in eliminating infected cells and modulating adaptive immunity, also showed distinctive patterns. COVID-19 patients had lower median frequencies of CD56bright NK cells compared to Control-LS individuals (0.30; IQR: 0.20-0.42 vs. 0.38; IQR: 0.29-0.57, P = 0.014), suggesting increased immune exhaustion (**Figure 3C**). Moreover, NK cells from COVID-19 patients exhibited increased Granzyme B expression, with CD56bright NK cells showing a median of 87.41% (IQR: 82.78-92.34, P<0.0001), which was 10% higher than that observed in Control-LS individuals. Linear regression analyses indicated no significant influence of sex, age, diabetes mellitus, or hypertension on these findings (P-values > 0.20). In addition, a slight reduction in % NK Granzyme B+ is shown in COVID-19 women compared to Control-LS women (P = 0.0094) (**Supplementary Figure S11C**).

Overall, these findings delineate a distinct immunological profile characterized by plasmablast expansion, reduced pDC and NK cell frequencies, and heightened markers of immune exhaustion in COVID-19 patients compared to low-susceptible controls.

A good cellular response is essential to fight against any infection. This is driven by T cells, particularly CD4+ T helper cells. Upon pathogen stimulation, naive CD4 cells differentiate into T helper cell subsets that coordinate the immune response, including pathogen-specific helper cytotoxic T cells (CD8+) and stimulation of B cells to generate high-affinity antibody responses to the pathogen. In addition, the age-associated accumulation of senescent cells in tissues is one of the driving causes of mammalian aging and age-related disease.

**Figure 4:**
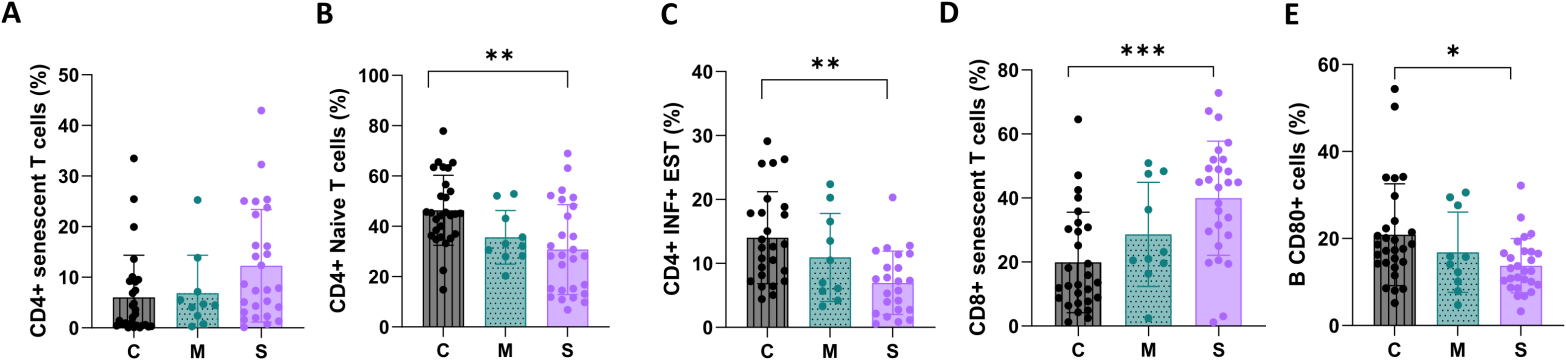
Characterization of senescence, naïve T cell subpopulation and functional response to antigens. Patients are divided according to the severity of their infection: Controls_LS (C) are in gray with vertical line pattern, mild COVID19+ cases (M) are in green with dots pattern and moderate-severe COVID19+ cases (S) are in purple with no fill pattern. Data were analyzed by one-way ANOVA, mean ± SEM (*p < 0.05, **p < 0.01, ***p< 0.005, ****p< 0.0001). Characterization of CD4+ T cells **(A–C)**; CD8+ T cells **(D)** and B cells **(E)**.

In our results, patients with SARS-CoV2 infection revealed a decrease in naïve CD4+ cells, as well as an increase in cellular senescence of both CD4+ and CD8+ T cells. Similarly, there is a decline in Th1 responsiveness, as well as a low capacity to respond to SARS-CoV2 neo-antigen. This correlates with the age of these COVID19+ patients, with a median age of 65 y-o compared to the Controls-LS of 43 y-o (p<0.001) (**Figure 4**).

## Discussion

In this study, we aimed to elucidate the role of different immunological markers in determining possible susceptibility to SARS-CoV-2 infection by analyzing a population of HCWs with repeated exposures to the virus but no evidence of infection, alongside individuals with varying degrees of disease severity, from mild to moderate-severe symptoms. We comprehensively characterized the function and phenotype of key immune cell populations involved in COVID-19 pathogenesis.

Consistent with prior reports, we found that plasmablasts were one of the first B-cell populations to expand in response to acute SARS-CoV-2 infection, potentially contributing to dysregulated immune responses. Plasmablasts are low-affinity antibody-secreting cells that proliferate and eventually mature into functional plasma cells capable of high-affinity antibody production. Their rapid expansion during acute infection has been noted in SARS-CoV-2 patients (10).

The elevated presence of plasmablasts in both mild and moderate-severe COVID-19 cases suggests that innate B-cell responses could contribute to the production of antibodies. Neutralizing antibodies (NAbs) are critical for protective immunity against SARS-CoV-2, as they block viral entry into host cells and correlate strongly with immune protection from symptomatic infection (17, 18). In our study, we observed increased titers of NAbs in COVID-19 patients, correlating with elevated plasmablast frequencies. This indicates an active, albeit potentially dysregulated, humoral response during the early stages of infection.

Our findings on plasmacytoid dendritic cells (pDCs) align with existing literature. pDCs are known for their potent antiviral capabilities, primarily producing type I interferons (16). Their numbers decreased significantly in COVID-19 patients compared to controls, likely contributing to impaired antiviral immunity. Previous studies have shown that pDCs produce high levels of type I interferons shortly after viral sensing, but this capacity is rapidly lost, a phenomenon described as “pDC exhaustion” (19, 20).

In our cohort, HCWs had significantly higher pDC frequencies than patients, suggesting that pDC-mediated interferon responses may contribute to generate protection against SARS-CoV-2 infection. The correlation between pDC depletion and disease severity was also supported by Liu et al. (21), who reported increased pDC apoptosis in severe cases (21). This apoptosis likely impairs the production of type I interferons, weakening antiviral defenses and contributing to uncontrolled viral replication and inflammation in severe COVID-19 cases. Notably, while pDCs are typically higher in women due to estrogen-driven TLR7 activity (22, 23), our study did not observe significant sex-based differences, indicating that other factors may modulate pDC dynamics in SARS-CoV-2 infection.

Another critical component of the immune response highlighted in our study is the role of Natural Killer (NK) cells, which are key players in antiviral immunity (24). NK cells can be divided into cytokine-producing CD56bright cells and cytotoxic CD56dim cells (25, 26). Although there is limited direct evidence of their function in SARS-CoV-2 infection, our study and others suggest that NK cells contribute to the immune response by directly targeting infected cells and modulating adaptive immunity (27, 28).

Interestingly, we found lower levels of CD56bright NK cells in COVID-19 patients compared to controls, indicating potential NK cell exhaustion or impaired function (29). This exhaustion may reduce the production of cytokines essential for modulating the adaptive immune response and impair the direct cytotoxic activity needed to eliminate infected cells, ultimately weakening the antiviral immune defense. This observation aligns with the findings by Maucourant et al., who reported no significant changes in NK cell percentages but noted functional alterations (30). The higher frequency of NK cells in HCWs may contribute to their resilience against infection, emphasizing the importance of NK cell-mediated antiviral defense.

Finally, aging leads to the decline of immunity, rendering the elderly susceptible to infection and disease (31). This decline in immune function, known as immunosenescence, is defined by disrupted CD4+ to CD8+ T cell ratios and chronic low-level inflammation, and contributes to the increased morbidity and mortality in older adults. As presented in this report, the age of patients affected by COVID19 is strongly correlated with decreased immune responsiveness due to reduced naïve and enhanced T-cell-associated senescence.

A major strength of our study is the analysis of a unique population: unvaccinated HCWs with low susceptibility to infection naive to SARS-CoV-2. This population no longer exists, making our findings particularly valuable for understanding immune mechanisms in the absence of vaccine-induced immunity.

However, several limitations must be acknowledged. The relatively small sample size, particularly in the mild group, and cross-sectional design may reduce statistical power and limit our ability to draw definitive conclusions about the temporal dynamics of immune responses. The recruitment of severely affected COVID-19 patients under strict clinical and biosafety conditions posed significant logistical challenges, which contributed to the limited cohort size. Therefore, our findings should be interpreted as exploratory and hypothesis-generating. Despite this, the consistent trends observed in immune cell frequencies and functional profiles across individuals support the robustness of the findings. Future studies involving larger and longitudinal studies are needed to better understand how these immune markers evolve throughout infection and recovery, addressing gaps such as the temporal progression of immune exhaustion, the durability of protective responses, and the interplay between innate and adaptive immunity over time.

In conclusion, the SARS-CoV-2 pandemic has highlighted significant gaps in our understanding of immune responses to respiratory viruses. Our findings suggest that dysregulated immune responses, including plasmablast expansion, reduced pDC, and impaired NK cell function, are potential critical factors in COVID-19 susceptibility. As previously reported, pDCs are a major source of type I interferons. Several studies have reported a reduction in circulating pDCs during acute SARS-CoV-2 infection, which may result in impaired interferon-mediated antiviral signaling, potentially favoring viral persistence and dysregulated immune activation. Similarly, NK cells exhibit alterations in both frequency and phenotype in COVID-19 patients. The interplay between pDC-derived interferons and NK cell activation is well established, as interferon signaling enhances NK cytotoxicity and cytokine production. Therefore, concomitant decreases in pDC and NK cell frequencies could synergistically impair innate antiviral defense mechanisms, contributing to disease severity and variability in susceptibility. In contrast, strong pDC and CD56bright NK responses may help protect against infection, as seen in repeatedly exposed but uninfected HCWs. Larger longitudinal studies are needed to elucidate the roles of these immune cells in COVID-19 susceptibility and progression, leveraging these insights to inform the design of targeted preventive and therapeutic strategies, including vaccines and immunomodulatory treatments.

## Data Availability

The raw data supporting the conclusions of this article will be made available by the authors, without undue reservation.
