# Supplementary material for: Contrasting immune responses in COVID-19: insights from healthcare workers and infected patients on plasmablast, pDC, and NK cell dynamics
Source: Front Immunol. 2026 Jan 7;16:1693903. doi: 10.3389/fimmu.2025.1693903 (PMC12819764; doi:10.3389/fimmu.2025.1693903)
Supplement: Supplementary file 1 [file Table1.docx]

**Supplementary Figures**


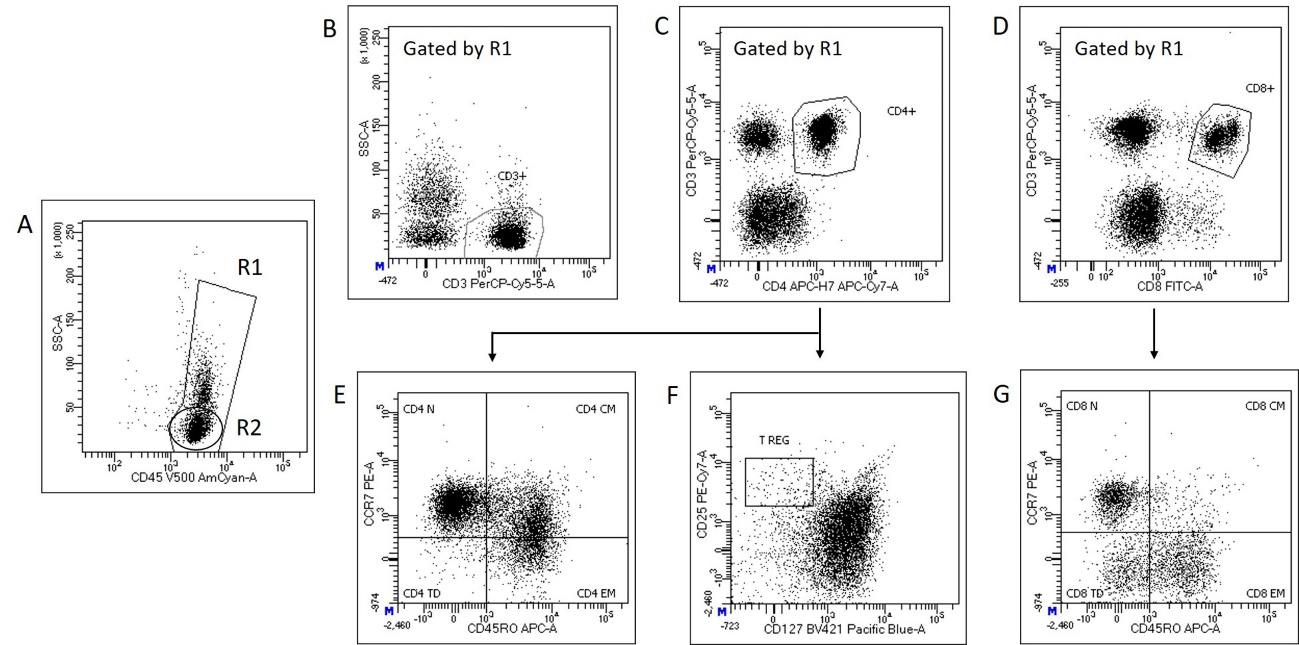


**Figure S1.** Gating strategy for T-cells. Total events were first gated to exclude debris, duplets and non-viable cells using 7-AAD, main region was set around viable cells expressing intermediate to high CD45 with low to intermediate side scater to select PBMC (A, gate R1) and total lymphocytes (A, gate R2). T-cells were identified as CD45+ and CD3+ cells (B). T helper cells were defined as CD4+ CD3+ cells (C), and their subpopulations were categorised as naïve (N; CCR7+, CD45RO-), central memory (CM; CCR7+, CD45RO+), effector memory (EM; CCR7-, CD45RO+), and terminally differentiated (TD; CCR7-, CD45RO-; E). Regulatory T-cells were defined as CD4+ CD3+, CD25high, CD127- cells; F). T cytotoxic cells were defined as CD8+ CD3+ cells (D), and their subpopulations were categorised as naïve (N; CCR7+, CD45RO-), central memory (CM; CCR7+, CD45RO+), effector memory (EM; CCR7-,CD45RO+), and terminally differentiated (TD; CCR7-,CD45RO-; G).


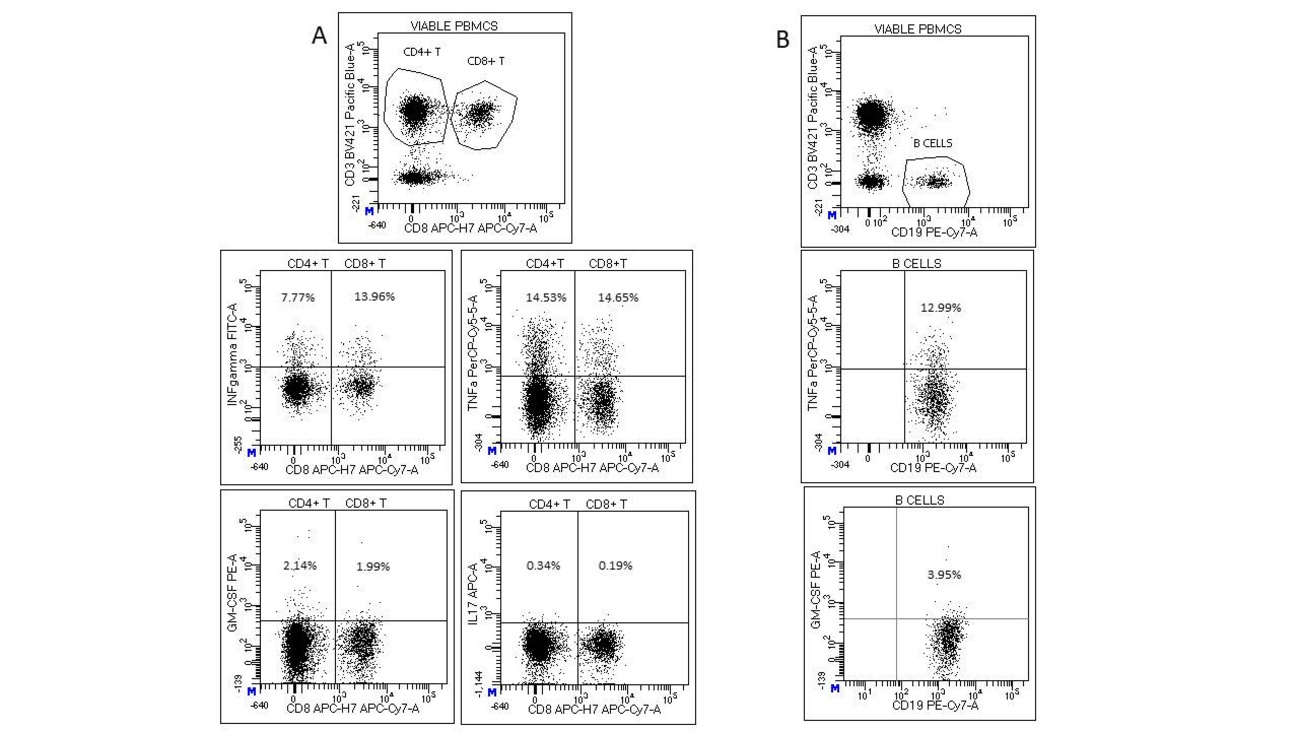


**Figure S2.** Representative images of phorbol-myristate-acetate (PMA) and ionomycin stimulated CD4+ and CD8+ T lymphocytes producing TNFα, interferon gamma (IFNγ), granulocyte macrophage-colony stimulating factor (GM-CSF) and IL17. Percentages are referred to total CD4+ or CD8+ T cells, respectively. (C) Representative images of PMA and ionomycin stimulated B lymphocytes producing TNFα and GM-CSF. Percentages are referred to total CD19+ B cells.

**Figure S3.** Distribution of general T cell subpopulation. Patients are divided according to the severity of their infection: Controls_LS (C) are in grey, mild COVID19+ cases (M) are in green, and moderate-severe COVID19+ cases (S) are in purple. Data were analysed by one-way ANOVA, mean ± SEM (*p < 0.05, **p < 0.01, ***p< 0.005, ****p< 0.0001).

**Figure S4.** Distribution of T cell maturation subpopulation. Patients are divided according to the severity of their infection: Controls_LS (C) are in grey, mild COVID19+ cases (M) are in green, and moderate-severe COVID19+ cases (S) are in purple. Data were analysed by one-way ANOVA, mean ± SEM (*p < 0.05, **p < 0.01, ***p< 0.005, ****p< 0.0001).

**Figure S5.** Distribution of T cell senescence subpopulation. Patients are divided according to the severity of their infection: Controls_LS (C) are in grey, mild COVID19+ cases (M) are in green, and moderate-severe COVID19+ cases (S) are in purple. Data were analysed by one-way ANOVA, mean ± SEM (*p < 0.05, **p < 0.01, ***p< 0.005, ****p< 0.0001).

**Figure S6.** Distribution of monocyte subpopulation. Patients are divided according to the severity of their infection: Controls_LS (C) are in grey, mild COVID19+ cases (M) are in green, and moderate-severe COVID19+ cases (S) are in purple. Data were analysed by one-way ANOVA, mean ± SEM (*p < 0.05, **p < 0.01, ***p< 0.005, ****p< 0.0001).

**Figure S7.** Distribution of dendritic cells (plasmocytoid and myeloid) subpopulation. Patients are divided according to the severity of their infection: Controls_LS (C) are in grey, mild COVID19+ cases (M) are in green, and moderate-severe COVID19+ cases (S) are in purple. Data were analysed by one-way ANOVA, mean ± SEM (*p < 0.05, **p < 0.01, ***p< 0.005, ****p< 0.0001).

**Figure S8.** Distribution of NK cell subpopulation. Patients are divided according to the severity of their infection: Controls_LS (C) are in grey, mild COVID19+ cases (M) are in green, and moderate-severe COVID19+ cases (S) are in purple. Data were analysed by one-way ANOVA, mean ± SEM (*p < 0.05, **p < 0.01, ***p< 0.005, ****p< 0.0001).

**Figure S9.** Distribution of B cell subpopulation. Patients are divided according to the severity of their infection: Controls_LS (C) are in grey, mild COVID19+ cases (M) are in green, and moderate-severe COVID19+ cases (S) are in purple. Data were analysed by one-way ANOVA, mean ± SEM (*p < 0.05, **p < 0.01, ***p< 0.005, ****p< 0.0001).

**Figure S10.** Distribution of Lymphocytes CK inflammatory subpopulation. Patients are divided according to the severity of their infection: Controls_LS (C) are in grey, mild COVID19+ cases (M) are in green, and moderate-severe COVID19+ cases (S) are in purple. Data were analysed by one-way ANOVA, mean ± SEM (*p < 0.05, **p < 0.01, ***p< 0.005, ****p< 0.0001).


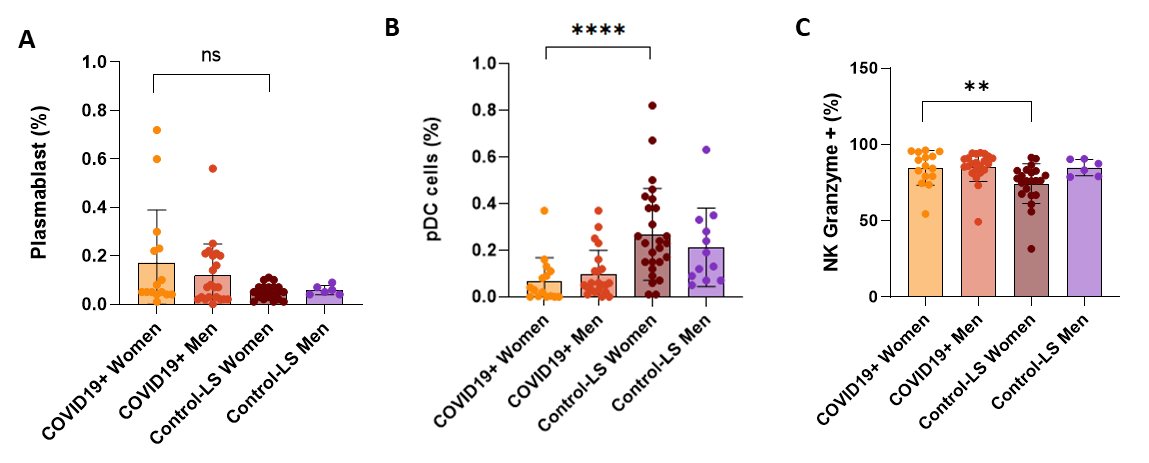


**Figure S11. (A)** Percentage of Plasmablast, (**B**) Plasmacytoid Dendritic cells (pDC) and (**C**) NK Granzima+ according to the sex of the individuals analysed. Patients are divided according to the presence of infection: Controls_LS individuals with no COVID-19 disease and COVID19+ patients.


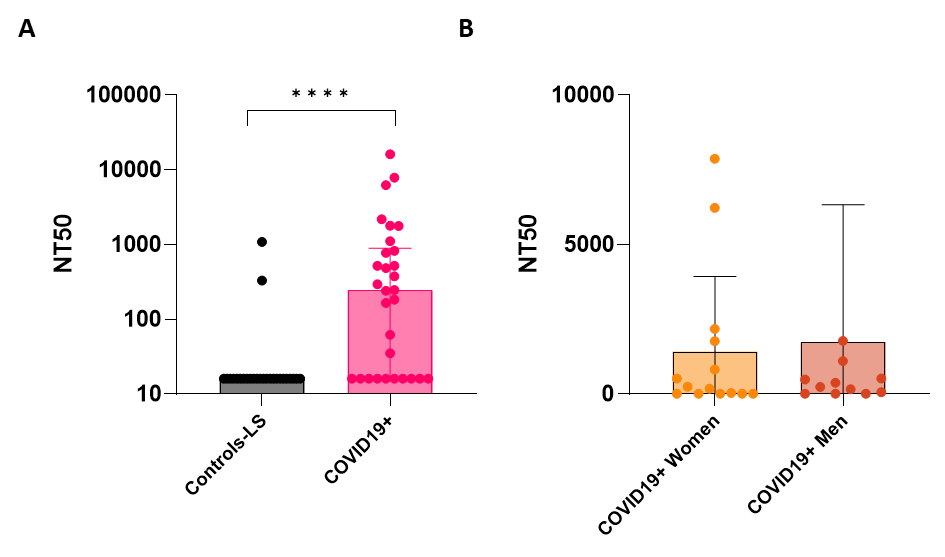


**Figure S12. (A)** Neutralization titers against SARS-CoV-2 spike pseudotyped viruses in COVID-19 susceptible and COVID-19 non-susceptible individuals. **(B)** Neutralization titers according to the sex of the individuals analysed. Statistical analysis: U Mann Whitney test, ****p < 0.0001. Plasma samples were preincubated with pseudoviruses at 37°C for 1 h. Sera and virus mixture were then incubated with VeroE6 cells for 48 h. Luciferase was measured to assess infection. NT50 was summarized as median and interquartile range.

**Figure S13.** Figure 4. Correlation between plasmablast frequency and SARS-CoV-2 neutralizing antibody titers. Scatter plot showing the relationship between plasmablast frequency (% of B cells) and SARS-CoV-2 neutralizing antibody titers (NT₅₀, log₁₀-transformed) in COVID-19 patients. Each dot represents one individual. The black line represents the linear regression fit, and the correlation was analyzed using Spearman’s rank correlation test (r = 0.34, p = 0.045) (*p < 0.05, **p < 0.01, ***p< 0.005, ****p< 0.0001). A positive correlation indicates that higher plasmablast frequencies are associated with greater neutralizing activity, suggesting that plasmablast expansion reflects the magnitude of the humoral response during acute infection.
